# Supplementary material for: HDAC Inhibition with Valproate Improves Direct Cytotoxicity of Monocytes against Mesothelioma Tumor Cells
Source: Cancers (Basel). 2022 Apr 26;14(9):2164. doi: 10.3390/cancers14092164 (PMC9100202; doi:10.3390/cancers14092164)
Supplement: Supplementary file 1 [file cancers-14-02164-s001.zip › cancers-1660468-supplementary-final/Supplementary files/cancers-1660468-suppl-proofread.pdf]

cancers

MDPI

Article

HDAC Inhibition with Valproate Improves Direct Cytotoxicity of Monocytes against Mesothelioma Tumor Cells

Clotilde Hoyos, Alexis Fontaine, Jean-Rock Jacques, Vincent Heinen, Renaud Louis, Bernard Duysinx, Arnaud Scherpereel, Eric Wasielewski, Majeed Jamakhani, Malik Hamaidia and Luc Willems

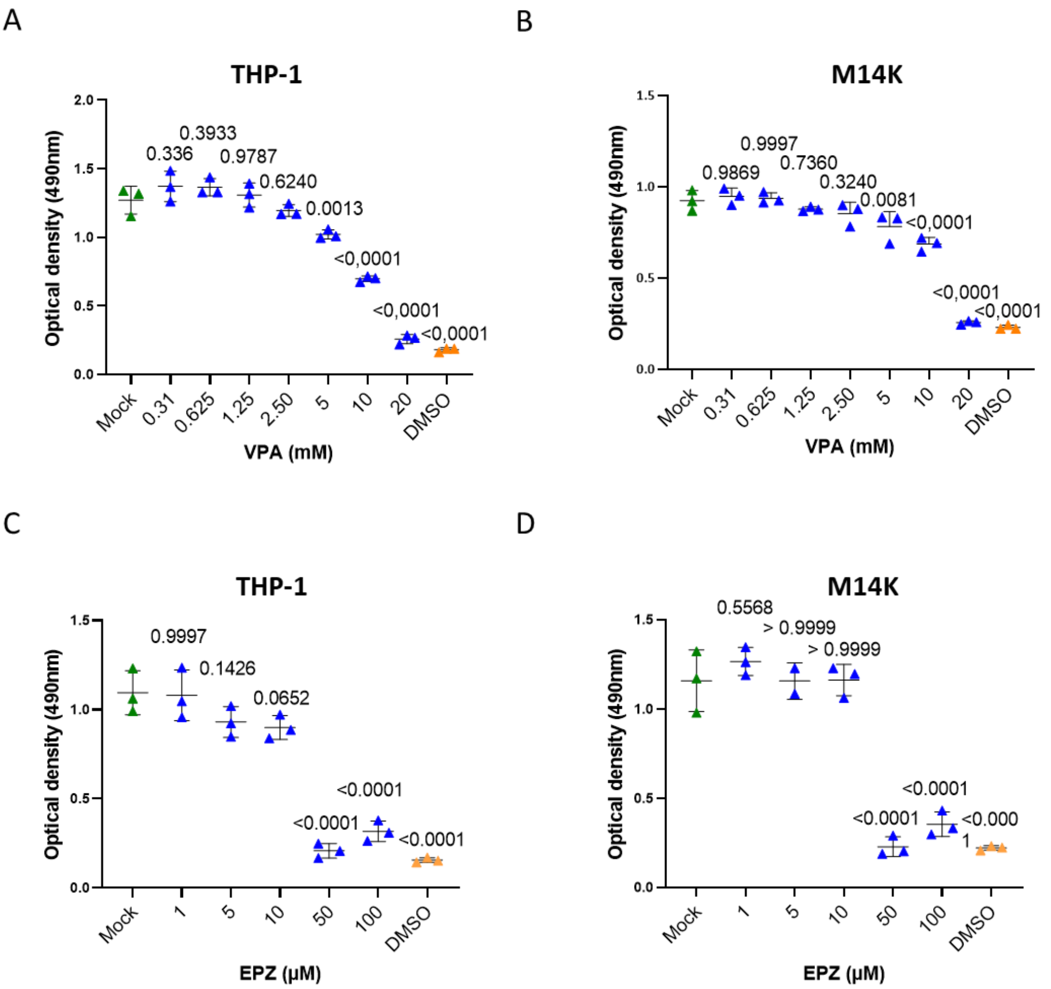

Figure S1. Dose-response of VPA and EPZ on metabolic activity of THP-1 and M14K cells. THP-1 (**A**) and M14K cells (**B**) were cultured with increasing concentrations of VPA (0, 0.31, 0.625, 1.25, 2.5, 5, 10 and 20 mM). THP-1 (**C**) and M14K cells (**D**) were incubated with increasing concentrations of EPZ (0, 1, 5, 10, 50 and 100 µM). After 48 hours, activity of NAD(P)H-dependent dehydrogenases was measured using the MTS assay. Treatment of cells with DMSO (10 %) was used as positive control. Data are presented as the means of three independent experiments ± standard deviations. Statistical significance was evaluated using a one-way ANOVA test with Dunnett's multiple comparison test.

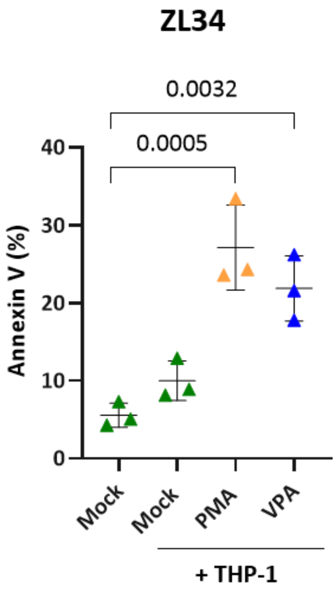

Figure S2. Direct cytotoxicity of THP-1 in contact with mesothelioma ZL34 cells measured by flow cytometry. THP-1 cells were cultured in presence of 16.2 µM of PMA or 2.5 mM of VPA during 48 hours. After washing the medium, THP-1 were cocultured with CFSE-labeled ZL34 cells at a 10/1 ratio during 48 hours. Annexin V^{+} ZL34 cells were counted by flow cytometry. Data (%) are presented as the means of three independent experiments ± standard deviations. Statistical significance was evaluated using a one-way ANOVA with Tukey's multiple comparison test.

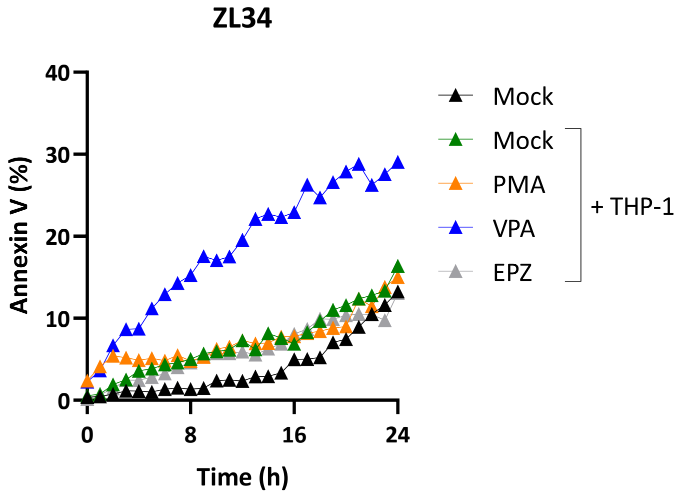

Figure S3. Direct cytotoxicity of THP-1 in contact with mesothelioma ZL34 cells analyzed by Incucyte THP-1 cells were cultured in presence of 16.2 µM of PMA, 2.5 mM of VPA or 10 µM of EPZ during 48 hours. After washing the medium, THP-1 were cocultured with CFSE-labeled ZL34 cells at a 1/1 ratio during 24 hours. The percentage of annexin V positive CFSE-labeled ZL34 cells was calculated based on Incucyte imaging data. Data (%) are presented as the means of three independent experiments ± standard deviations. Statistical significance was evaluated using a one-way ANOVA with Tukey's multiple comparison test.

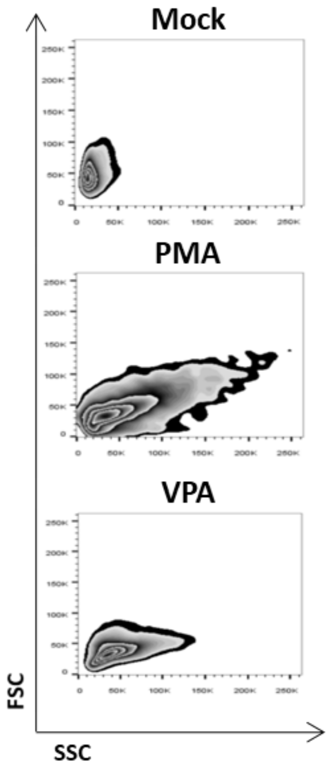

Figure S4. Effect of PMA and VPA on THP-1 cell size and granularity THP-1 cells were cultured in presence of 16.2 µM of PMA or 2.5 mM of VPA during 48 hours and analyzed by flow cytometry. Forward scatter (FSC) and side scatter (SSC) profiles allow for the discrimination of cells by size and granularity, respectively.

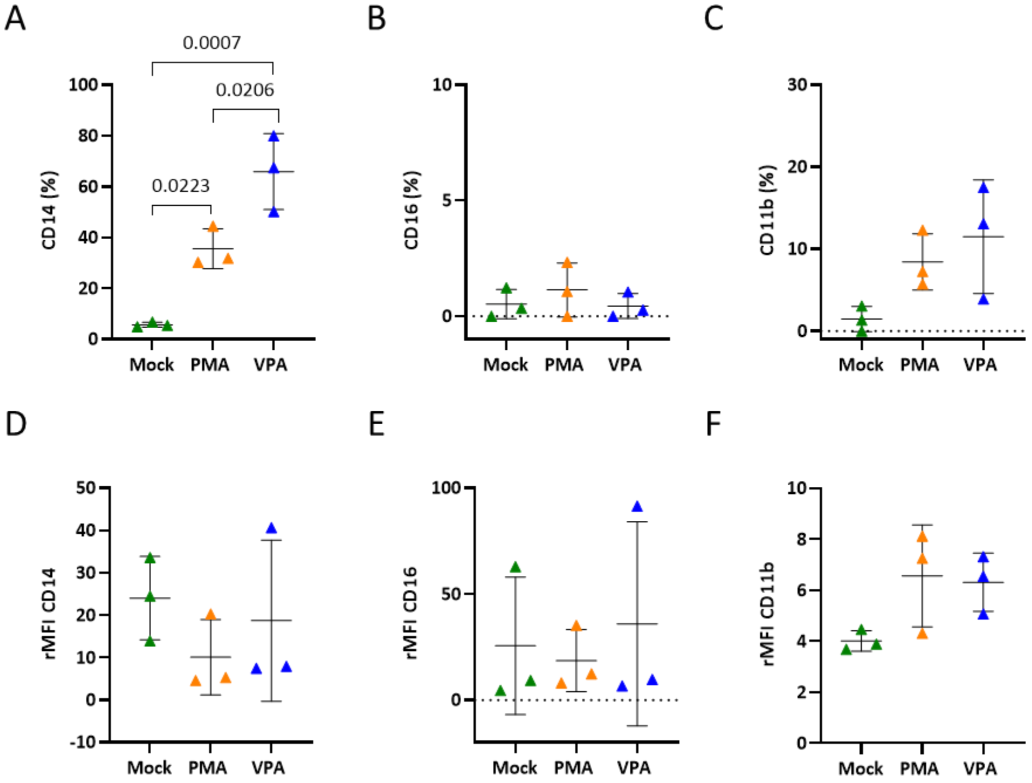

Figure S5. Effect of PMA and VPA on expression of CD14, CD16 and CD11b by THP-1 cellsTHP-1 cells (1x10[6] cells in a 6 well plate) were cultured in absence (mock) or in presence of 16.2 µM of PMA or 2.5 mM of VPA for 48 hours. Cells were labeled with antibodies directed against CD16 (*Immunotools*) or CD11b (*Immunotools*) followed by Alexa Fluor 647 conjugates (*Invitrogen*) and anti-CD14-PE (*Beckman Coulter*). After recording events by flow cytometry (BD FACSCanto, *BD Biosciences*), data were analyzed with FlowJo v10 software (*BD Biosciences*). Data were expressed in percentages of fluorescent cells (**A-C**) or in ratio of mean of fluorescence intensity (rMFI) (**D-E**). Data (%) are presented as the means of three independent experiments ± standard deviations. Statistical significance was evaluated using a one-way ANOVA with Tukey's multiple comparison test.

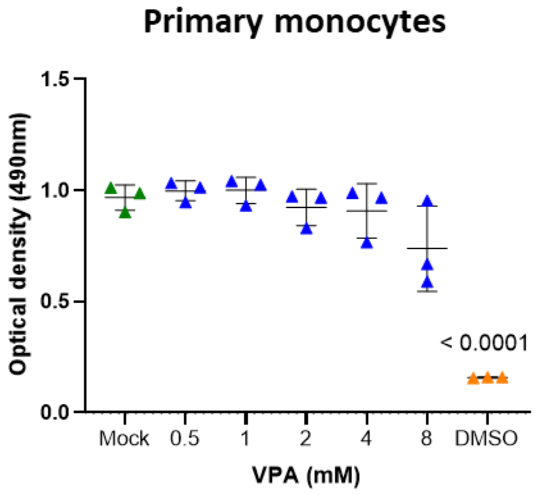

Figure S6. Dose-response of VPA on metabolic activity of primary monocytes After isolation, primary monocytes were cultured in presence of increasing concentrations of VPA (0, 0.5, 1, 2, 4 and 8 mM) during 24 hours. Cell viability activity was measured using the MTS assay. Treatment of primary monocytes with DMSO (10 %) was used as positive control. Data (optical densities at 490 nm) are presented as the means of three independent experiments ± standard deviations. Statistical significance was evaluated using a one-way ANOVA test with Dunnett's multiple comparison test.

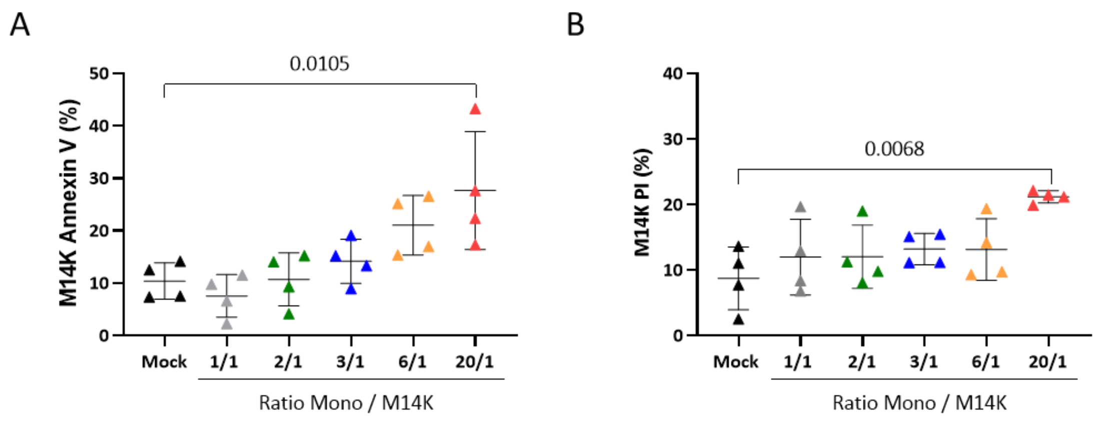

Figure S7. Direct cytotoxicity of blood-derived monocytes in contact with mesothelioma M14K cells at different ratios. After isolation, primary monocytes were cocultured with CFSE-labeled M14K at different monocyte/M14K cell ratios 1/1 (3x10[3]/3x10[3]), 2/1 (6x10[3]/3x10[3]), 3/1 (9x10[3]/3x10[3]), 6/1 (18x10[3]/3x10[3]) and 20/1 (6x10[4]/3x10[3]). Cell cultures were recorded by Incucyte in presence of annexin V (**A**) or propidium iodide (**B**) during 24 hours. The percentages of annexin V (**A**) or propidium iodide (**B**) positive CFSE-labeled M14K cells were retrieved after 24 hours from the Incucyte

data sets. The means ± standard deviations result from four independent experiments. Statistical significance was evaluated using a one-way ANOVA with Tukey's multiple comparison test.

Table S1. Results of DGEA between conditions D1 and D0.

Table S2. Results of DGEA between conditions D1+VPA and D1.

Table S3. Results of DGEA between conditions D1+VPA and D0.

Table S4. Gene ontology classification and pathway enrichment analysis.

Video S1. Direct cytotoxicity of a THP-1 monocyte towards CFSE-labeled M14K revealed by annexin V labeling. THP-1 monocytes were cocultured with CFSE-labeled M14K cells (in green) at a 1/1 ratio during 48 h in the presence of annexin V-APC (in red). Cells were maintained at 37 °C in a humidified atmosphere containing 5% CO_2. The video was recorded by Incucyte time-lapse microscopy.

Video S2. Direct cytotoxicity of a THP-1 monocyte towards CFSE-labeled M14K in the presence of propidium iodide. THP-1 monocytes were cocultured with CFSE-labeled M14K cells (in green) at a 1/1 ratio during 48 h in the presence of propidium iodide (in red). Cells were maintained at 37 °C in a humidified atmosphere containing 5% CO_2. The video was recorded by Incucyte time-lapse microscopy.

Video S3. Direct interaction of a blood-derived monocyte towards CFSE-labeled M14K. M14K cells were labeled with CFSE, incubated for 4 h, and then cocultured with freshly isolated monocytes at a ratio of 4/1. After 24 h of coculture on coverslips, cells were labeled with an anti-CD33 primary antibody followed by Alexa fluor 647 conjugate. Cells were further fixed with PFA 4%, stained with DAPI, and mounted on slides before analysis with a HR confocal microscope. Images were computed by Imaris. Representative interaction between a CFSE-positive M14K cell (in green) and CD33-labeled monocyte (in red) is shown. Nuclei fragmentation is revealed by the DAPI staining (in blue).

Video S4. Direct cytotoxicity of a blood-derived monocyte toward CFSE-labeled M14K. See legend of Video 3 for experimental details.

Video S5. Direct cytotoxicity of a blood-derived monocyte towards CFSE-labeled M14K in the presence of annexin V. M14K cells were labeled with CFSE (in green), incubated for 4 h, and then cocultured with freshly isolated monocytes at a ratio of 20/1 in the presence of annexin V APC (in red) during 24 h. Cells were maintained at 37 °C in a humidified atmosphere containing 5% CO_2. The video was recorded by Incucyte time-lapse microscopy.

Video S6. Direct cytotoxicity of a blood-derived monocyte towards CFSE-labeled M14K in the presence of propidium iodide. M14K cells were labeled with CFSE (in green), incubated for 4 h, and then cocultured with freshly isolated monocytes at a ratio of 20/1 in the presence of propidium iodide (in red) during 24 h. Cells were maintained at 37 °C in a humidified atmosphere containing 5% CO_2. The video was recorded by Incucyte time-lapse microscopy.
